# Exponential Consensus of Multi-Agent Systems under Event-Triggered Impulsive Control with Actuation Delays

**DOI:** 10.3390/e25060877

**Published:** 2023-05-30

**Authors:** Jian Zhang, Shiguo Peng

**Affiliations:** School of Automation, Guangdong University of Technology, Guangzhou 510006, China; 2112104036@mail2.gdut.edu.cn

**Keywords:** actuation delays, event-triggered mechanism, impulsive control, multi-agent system, exponential consensus

## Abstract

This paper investigates the exponential consensus problem for a class of nonlinear leader-following multi-agent systems using impulsive control, where impulses are generated by the event-triggered mechanism and are subjected to actuation delays. It is proved that Zeno behavior can be avoided, and by employing the linear matrix inequality technique, some sufficient conditions for realizing exponential consensus of the considered system are derived. Actuation delay is an important factor affecting the consensus of the system, and our results show that increasing the actuation delay can enlarge the lower bound of the triggering interval, while it harms the consensus. To demonstrate the validity of the obtained results, a numerical example is provided.

## 1. Introduction

A multi-agent system (MAS) is comprised of a flock of agents that can communicate over a network and engage in collaborative behaviors, which can effectively utilize system resources. Among these behaviors, achieving consensus is a key issue, and it has attracted the attention of many scholars over the past two decades. The so-called consensus of a MAS [1,2,3,4] can generally be described as all agents reaching or converging to the same value. For a leader-following MAS, consensus means that the followers are able to follow the leader effectively, such as the research on the consensus of uncertain network systems [5], and the design of observers to achieve the consensus of multi-agent systems with delays [6]. Compared to a traditional leaderless system, a leader-following MAS is more robust and capable of performing more complex and collaborative tasks. Hence, the latter is more general and more valuable to study. Furthermore, it has been used in many real-life production situations, such as mobile robots, cooperative guidance, and ship course-keeping [7,8,9].

In earlier works [10], agents had to continuously send their current state to controllers in order to achieve consensus, leading to significant consumption of communication resources. However, this was later found to be unnecessary. To alleviate the pressure on traffic load, several methods have been established as effective. For instance, ref. [11] discusses the adept use of the logarithmic quantizer and the weighted try-once-discard protocol, all based on sliding mode control, to enhance the utilization of system resources. Moreover, ref. [12] investigated the issue of leader-following consensus in a specific class of systems and devised three control schemes to minimize frequent communication between systems, thereby effectively avoiding resource wastage. Taking inspiration from these studies, this article employs event-triggered control and impulsive control to achieve the same objective.

Event-triggered control [13,14,15] and impulsive control [16,17,18]. Both methods update the interactive information at discrete points but differ in how to update. Event-triggered control updates the information when preset threshold conditions are met, but it still requires continuous control and leads to high costs. Impulsive control, on the other hand, updates the information at a prescribed frequency, but this method cannot adapt to the needs of the system and wastes resources [19].

Though these control methods have received considerable attention [20,21,22], they cannot make full use of resources in some sense with a single control method. To address this issue, a combination called event-triggered impulsive control has been introduced, which updates and controls the system at discrete instants only when necessary. For example, it has been used to achieve consensus both with and without external disturbance [23]; some conditions for leader-following consensus using linear matrix inequalities (LMIs) have been proposed under a distributed framework [24], and sufficient conditions for nonlinear stochastic system to reach exponential consensus under the effect of LMI-based event-triggered impulse control were presented in [25]. However, it should be noted that the above works only consider instantaneous impulses and ignore possible delays.

Time delays are common in nature and engineering. In recent years, delayed impulses have been extensively studied, where delays exist in the sampling and transmission of impulsive controllers. For instance, delayed impulses were studied for exponential stability in [26]. Later, it was extended to the consensus of a MAS, synchronization of complex networks, and some event-triggered cases. In [27], a novel hybrid impulse control protocol with actuation delays was designed, and a sufficient condition for the system to achieve average consensus was given. In [28], sufficient conditions for the controller to achieve global consensus under actuator saturation for switching topologies and time-varying delays were presented. In [29,30], two different event-triggered mechanisms based on Lyapunov functions were designed, and the stability of the system with delayed impulses was investigated, respectively.

However, due to the limitations of hardware response or computation speeds, actuators may require some time to execute, and these times are called actuation delays [31]. The presence of actuation delays can degrade system performance or even cause instability. Compared with delayed impulses, actuation delays mainly focus on the effects caused by delays after impulses, instead of using the past values. Nevertheless, there are still few studies on event-triggered control with actuation delays, and most of them are only asymptotically stable. In [32], a novel event-triggered impulsive mechanism (ETIM) without exhibiting Zeno behavior was designed, and conditions for the asymptotic stability of a class of nonlinear systems were derived. As important as delayed impulses, effects caused by actuation delays deserve further study but have received little attention so far.

Inspired by the above analysis, this paper investigates the exponential consensus of a class of nonlinear leader-following MASs with actuation delays under the designed ETIM. Our contributions can be summarized as follows:

- A novel ETIM is proposed, which can exclude Zeno behavior and realize the exponential consensus. With the designed ETIM, some LMI-based sufficient conditions are proposed to achieve the exponential consensus. Moreover, compared with the existing results, the threshold condition is only time-dependent, so the triggered information does not need to be memorized, which makes it easy to implement.
- Different actuation delays are considered in the system model, and it is more general and practical than [32], as delays are not always the same. Compared with [33], restrictions on the decay rate are not required. Since different actuation delays are considered in the impulsive control, the main challenge lies in the estimation of the error state at each triggering instant and the combination of the system dynamics and the designed event-triggered mechanism.
- In comparison to the system with a fixed actuation delay studied in [32], the system proposed in this paper is more versatile as it considers different actuation delays at each impulse instant. Moreover, by utilizing ETIM, our system is capable of achieving exponential consensus.

The remainder of this paper is organized as follows: Section 2 provides some preliminaries and describes the model. Section 3 presents the main results, including the exclusion of Zeno behavior and the LMIs-based sufficient conditions for consensus obtained using the proposed ETIM. Section 4 offers one numerical example to illustrate the effectiveness of the proposed results. Finally, Section 5 concludes the paper.

**Notation 1.**  *Let $R,R^{+},N^{+},R^{n},R^{n\times m}$ denote the set of real numbers, non-negative real numbers, positive integers, the n-dimensional Euclidean space, and all the n×m-dimensional real matrices, respectively. For any vector or real matrix Q, let $∥Q∥$ denote the Euclidean or induced norm. For any symmetrical matrix Q, $\lambda_{\max}\left(Q\right)$ stands for its maximum eigenvalue. diag{·} represents the diagonal matrix, and exp(·) denotes the exponential function. In addition, the paper will use* ⊗ *to represent Kronecker product. Let $I_{N}=\left\{1,2,\ldots ,N\right\}$ represent a finite set. Function $\alpha :R^{+}\to R^{+}$ belongs to class K if it is continuous, strictly increasing and α(0)=0.*

## 2. Preliminaries

### 2.1. Graph Notions

A leader-following MAS consisting of one leader and *N* followers can be represented by a topology graph G=(V,E,A), where the vertex set V is defined as $V=\{v_{0},v_{1},\ldots ,v_{N}\}$, and the edge set E⊆V×V. The subscript 0 denotes the leader, while the rest of the agents are followers. The neighbor index set of agent *i* is defined as $N_{i}=\left\{v_{j}\in V|\left(v_{j},v_{i}\right)\in E\right\}$, indicating the agents that are connected to agent *i*.

The adjacency matrix $A={\left[a_{ij}\right]}_{N\times N}$ is defined such that $a_{ij}>0$ if there exists an edge between agent *i* and agent *j*, and $a_{ij}=0$ otherwise. The Laplacian matrix $L={\left[l_{ij}\right]}_{N\times N}$ is defined as L=D−A, where $D=\mathrm{diag}\left\{\sum_{j\in N_{1}}a_{1j},\sum_{j\in N_{2}}a_{2j},\ldots ,\sum_{j\in N_{N}}a_{Nj}\right\}$ is the degree matrix. Moreover, if there exists a leader that sends a message directly to a follower agent, then a directed edge is present from the leader to the follower. In this case, the diagonal matrix $C=\mathrm{diag}\{c_{1},c_{2},\ldots c_{N}\}$ is defined such that $c_{i}>0$ if agent *i* is a follower receiving a message from the leader, and $c_{i}=0$ otherwise. Furthermore, let H=L+C.

### 2.2. Model Description

Consider a leader-following MAS consisting of one leader and *N* followers, where the dynamic of the leader is described as

(1) $${\dot{x}}_{0}\left(t\right)=Ax_{0}\left(t\right)+Bf\left(x_{0}\left(t\right)\right),$$

where $x_{0}\left(t\right)\in R^{n}$ is the state of leader, *A*, $B\in R^{n\times n}$ represent the system matrix, $f:R^{n}\to R^{n}$ satisfies f(0)=0. The dynamic of follower $i,\forall i\in I_{N}$ is given by

(2) $${\dot{x}}_{i}\left(t\right)=Ax_{i}\left(t\right)+Bf\left(x_{i}\left(t\right)\right)+u_{i}\left(t\right),$$

where $x_{i}\left(t\right)\in R^{n}$ is the state of follower *i*, $i\in I_{N}$, matrices A,B and the function *f* as given in (1). Furthermore, suppose that $t_{0}$ is the initial instant, while $x_{0}\left(t_{0}\right)$ and $x_{i}\left(t_{0}\right)$ are initial states of the leader and follower *i*, respectively. To reach the consensus of system (1) and (2), the impulsive controller $u_{i}\in R^{n}$ is designed as

(3) $$\begin{array}{cc} u_{i}\left(t\right)= & K\sum_{k=1}^{+\infty }\sum_{j\in N_{i}}[a_{ij}\left(x_{i}\left(t-\tau_{k}\right)-x_{j}\left(t-\tau_{k}\right)\right)\\ \; & +c_{i}\left(x_{i}\left(t-\tau_{k}\right)-x_{0}\left(t-\tau_{k}\right)\right)]\delta \left(t-d_{k}\right), \end{array}$$

where $K\in R^{n\times n}$ is the impulsive gain matrix, δ(t) is the Dirac function representing the action of the impulse; the time sequence determined by the ETIM can be defined as $\left\{t_{k},k\in N^{+}\right\}$; $\tau_{k}\in \left[0,\tau_{\sup}\right]$ denotes the bounded actuation delays under different control instants $t_{k}$, where $\tau_{\sup}=\sup_{k\in N^{+}}\left\{\tau_{k}\right\}$. Let $\left\{d_{k},k\in N^{+}\right\}$ be the delayed impulsive sequence with $d_{k}=t_{k}+\tau_{k}$ and set $d_{0}=t_{0}$. In addition, assume that the system is right-continuous at each impulsive instant, $x_{i}\left(d_{k}^{+}\right)=x_{i}\left(d_{k}\right)$.

**Remark 1.**  *Impulsive control is a widely used method for achieving consensus in MASs due to its simplicity and robustness. However, many existing studies ignore the fact that actuators require a certain amount of time to execute, which is described as $\tau_{k}$ in* (3). *However, the impact of time delay on the system is often not to be underestimated. On the other hand, impulses subjected to actuation delays are not the same as delayed impulses discussed in [26], although they share some similarities. In the former, information is collected at time $t_{k}$ and the impulse is executed at time $d_{k}$, while in the latter, information from a previous instant $r_{k}\in \left[t_{k-1},t_{k}\right)$ is used to calculate at time $t_{k}$.*

Define the error state $e_{i}\left(t\right)=x_{i}\left(t\right)-x_{0}\left(t\right),i\in I_{N}$ and $g\left(e_{i}\left(t\right)\right)=f\left(x_{i}\left(t\right)\right)-f\left(x_{0}\left(t\right)\right)$. Further, using the Kronecker product, according to (1)–(3), the error system can be described as follows.

(4) $$\left\{\begin{array}{c} \dot{e}\left(t\right)=\left(I_{N}⊗A\right)e\left(t\right)+\left(I_{N}⊗B\right)G\left(e\left(t\right)\right),t\geq t_{0},t\neq d_{k},\\ ∆e\left(d_{k}\right)=\left(H⊗K\right)e\left(t_{k}\right),\;t=d_{k}, \end{array}\right.$$

where $e\left(t\right)={\left[e_{1}^{T}\left(t\right),e_{2}^{T}\left(t\right),\ldots ,e_{N}^{T}\left(t\right)\right]}^{T}$ and $G\left(e(t)\right)={\left[g^{T}\left(e_{1}\left(t\right)\right),g^{T}\left(e_{2}\left(t\right)\right),\ldots ,g^{T}\left(e_{N}\left(t\right)\right)\right]}^{T}$.

### 2.3. Event-Triggered Mechanism

Considering the actuation delay, the ETIM is designed as follows:

(5) $$t_{k}^{*}=\inf\left\{t\geq d_{k-1}|V\left(e\left(t\right)\right)\geq a\exp\left(-b\left(t-t_{0}\right)\right)\right\},\;k\in N^{+},$$

where $a>V(e\left(t_{0}\right))$ and b>0 are some adjustable variables, and V(e(t)) is the Lyapunov function to be designed.

The threshold function of the ETIM (5) is given by $a\exp(-b\left(t-t_{0}\right))$. When the value of V(e(t)) exceeds or equals the threshold value $a\exp(-b\left(t-t_{0}\right))$, a new event is generated. It should be noted that the event-triggered mechanism takes into account the time delay $\tau_{k}$ that is required for actuators to execute the update, which distinguishes it from delayed impulses discussed in [26,33]. Moreover, it should be noted that due to the actuation delay, the ETIM (5) does not require monitoring during the interval $\left[t_{k},d_{k}\right)$.

**Figure 1:**
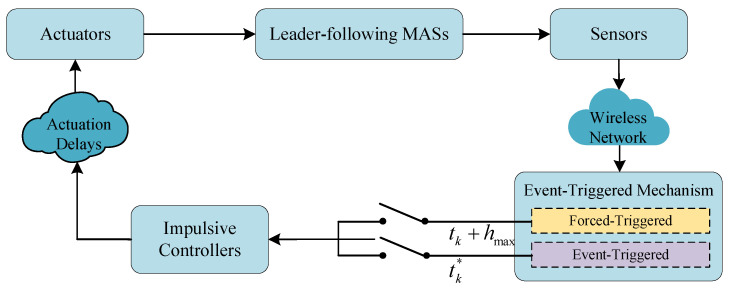
Mechanism for event-triggered impulsive control loop.

**Remark 2.**  *The closed-loop diagram illustrated in Figure 1 shows the closed system consisting of the leader-following MAS *(1) *and* (2)*, the impulsive controller* (3) *and the ETIM* (5). *The operation of the system starts with the sensors sampling relevant states of the system and sending them to the ETIM through wireless networks. If the sampled state values satisfy the condition $V\left(e\left(t\right)\right)\geq a\exp\left(-b\left(t-t_{0}\right)\right)$, then an event (an event-triggered impulse) will be generated. When an event occurs, the sampled state values will be transmitted to the impulsive controller, which generates a control signal. This control signal is then sent to the controller with some delays due to the restricted speed of the equipment. Finally, the system receives feedback from the actuator, and the process repeats.*

### 2.4. Definitions, Lemmas, and Assumptions

**Definition 1**  ([34])**.** *If there exist $\gamma_{1},\gamma_{2}>0$ such that $V\left(e\left(t\right)\right)\leq \gamma_{1}\exp\left(-\gamma_{2}\left(t-t_{0}\right)\right)V\left(e\left(t_{0}\right)\right)$, $\forall t\geq t_{0}$, then system *(1) *and* (2) *can achieve global exponential consensus.*

**Definition 2**  ([32])**.** *Under any ETIM, controlled system *(1) *and* (2) *exhibits Zeno behavior, if there exists a scale $T_{z}>0$ such that $t_{k}\leq T_{z}$ for any $k\in N^{+}$. Otherwise, there is no Zeno behavior.*

**Definition 3.**  *For a locally Lipschitz function $V:R^{Nn}\to R^{+}$, the upper right-hand Dini derivative of V along the solution of system *(4) *is defined by*

$$D^{+}V\left(e\left(t\right)\right)=\underset{\epsilon \to 0^{+}}{lim sup}\frac{1}{\epsilon }(V\left(e(t+\epsilon )\right)-V\left(e(t)\right)).$$

**Lemma 1**  ([35])**.** *For any $M\in R^{q\times l}$, $u\in R^{q}$, $v\in R^{l}$, and any positive definite matrix $G\in R^{l\times l}$, the following inequality holds:*

$$2u^{T}Mv\leq u^{T}MGM^{T}u+v^{T}G^{-1}v.$$

**Assumption A1.**  *The nonlinear function f in system *(1) *and* (2) *satisfies the Lipschitz condition, i.e., there exists a positive constant $L_{1}$, such that for any $y_{1},y_{2}\in R^{n}$,*

(6) $$∥f\left(y_{1}\right)-f\left(y_{2}\right)∥\leq L_{1}∥y_{1}-y_{2}∥.$$

**Assumption A2.**  *The communication topology of system *(1) *and* (2) *has a directed spanning tree, and the leader is the root node.*

## 3. Main Results

In this section, we first show that there is no Zeno behavior for system (1) and (2) under the designed ETIM (5). Then, sufficient conditions to ensure the exponential consensus are derived under the impulsive controller (3) and ETIM (5).

### 3.1. Exclusion of Zeno Behavior

**Theorem 1.**  *If there exists a locally Lipschitz continuous function $V:R^{Nn}\to R^{+}$ and some scalars $c_{1}>0$, $c_{2}\in \left(0,1\right)$ such that*

(7) $$\begin{array}{cc} \; & D^{+}V\left(e\left(t\right)\right)\leq c_{1}V\left(e\left(t\right)\right),\;t\neq d_{k},\;k\in N^{+}, \end{array}$$

(8) $$\begin{array}{cc} \; & V\left(e\left(t\right)\right)\leq c_{2}V\left(e\left(t_{k}\right)\right),\;t=d_{k},\;k\in N^{+}. \end{array}$$

*Then, the ETIM *(5) *does not exist in Zeno behavior. In addition, the triggering interval is bounded by*

$$\underset{k\in N^{+}}{\inf}\left\{t_{k+1}-t_{k}\right\}\geq -\frac{1}{b+c_{1}}\ln c_{2}.$$

**Proof.**  Assume that $\left\{t_{k},k\in N^{+}\right\}$ is generated by the ETIM (5). All elements in $\left\{t_{k},k\in N^{+}\right\}$ are generated by $t_{k}=t_{k}^{*}$. it follows from (7) that

(9) $$V\left(e\left(t_{k+1}\right)\right)\leq V\left(e\left(d_{k}\right)\right)\exp\left(c_{1}\left(t_{k+1}-d_{k}\right)\right),$$

and on the other hand, it follows from (5) and (8) that for any $k\in N^{+}$ has

(10) $$V\left(e\left(d_{k}\right)\right)\leq c_{2}V\left(e\left(t_{k}\right)\right)=c_{2}a\exp\left(-b\left(t_{k}-t_{0}\right)\right).$$

Combining with (9) and (10), one has

$$a\exp\left(-b\left(t_{k+1}-t_{0}\right)\right)\leq c_{2}a\exp\left(c_{1}\left(t_{k+1}-d_{k}\right)-b\left(t_{k}-t_{0}\right)\right),$$

which indicates that

(11) $$t_{k+1}-t_{k}\geq \tau_{k}-\frac{\ln c_{2}+b\tau_{k}}{b+c_{1}}.$$

Let $y\left(s\right)=s-\left(\ln c_{2}+bs\right)/\left(b+c_{1}\right)$. It can be checked that $\dot{y}\left(s\right)=c_{1}/\left(b+c_{1}\right)>0$, i.e., *y* is monotonically increasing. Hence, y(s)≥y(0) holds for any $s\in [0,\tau_{\sup}]$, and $\inf_{k\in N^{+}}\left\{t_{k+1}-t_{k}\right\}\geq -\frac{1}{b+c_{1}}\ln c_{2}>0$.

Hence, there is no Zeno behavior for system (1) and (2) under the designed ETIM (5). This completes the proof of Theorem 1. □

**Remark 3.**  *It can be checked that $-\ln c_{2}/\left(b+c_{1}\right)$ is derived by $\tau_{\inf}=\inf_{k\in N^{+}}\left\{\tau_{k}\right\}=0$, and thus, the ETIM *(5) *can avoid Zeno behavior when there is no actuation delays as usual. Additionally, the actuation delay plays an active role in excluding Zeno behavior from* (11)*. In this regard, the lower boundedness of $t_{k+1}-t_{k}$ can be enlarged as $\tau_{\inf}$ increases.*

**Remark 4.**  *Though Theorem 1 can ensure the exclusion of Zeno behavior, a special phenomenon will emerge when $d_{k}$ is the next triggering instant, i.e., $t_{k+1}=d_{k}$ for any $k\in N^{+}$. This situation will happen to the ETIM *(5) *because $V\left(e\left(d_{k}\right)\right)\geq a\exp\left(-b\left(d_{k}-t_{0}\right)\right)$ in this case. Hence, to avoid this phenomenon, one should further ensure that*

$$\begin{array}{cc} V(e\left(d_{k}\right))\; & \leq c_{2}V\left(e\left(t_{k}\right)\right)\leq c_{2}a\exp\left(-b\left(t_{k}-t_{0}\right)\right)\\ \; & \leq c_{2}a\exp\left(b\tau_{k}\right)\exp\left(-b(t_{k}+\tau_{k}-t_{0})\right)\\ \; & <a\exp\left(-b(t_{k}+\tau_{k}-t_{0})\right), \end{array}$$

*where the following condition is used in the last inequality:*

$$\tau_{\sup}<-\frac{1}{b}\ln c_{2}.$$

### 3.2. Consensus Analysis

**Theorem 2.**  *Let Assumptions 1 and 2 be satisfied. If there exist some scales $L_{1}>0,\mu >0,\eta >0,\rho_{0}\in \left(0,1\right),\rho \in \left(0,1\right)$ and some positive definite matrices $R_{1}\in R^{n\times n},R_{2}\in R^{n\times n}$ such that*

(12) $$\left[\begin{array}{cc} I_{N}⊗\left(A^{T}+A+R_{1}-\mu I_{n}\right) & L_{1}\left(I_{N}⊗B^{T}\right)\\ {}* & -I_{N}⊗R_{1} \end{array}\right]<0,$$

(13) $$\left[\begin{array}{ccc} I_{N}⊗ℵ & L_{1}\left(I_{N}⊗B^{T}\right) & I_{N}⊗A^{T}\\ {}* & -I_{N}⊗R_{2} & 0\\ {}* & * & -I_{N}⊗R_{2}^{-1} \end{array}\right]<0,$$

(14) $$\left[\begin{array}{cc} H^{T}⊗\left(K^{T}+K\right)+\left(1-\rho_{0}\right)\left(I_{N}⊗I_{n}\right) & H^{T}⊗K^{T}\\ {}* & -I_{N}⊗I_{n} \end{array}\right]<0,$$

(15) $$\tau_{sup}<\frac{-2\ln\rho }{\mu +b},$$

*where*

(16) $$\begin{array}{cc} \; & \rho =\sqrt{\rho_{0}}+\underset{k\in N^{+}}{\sup}\left\{\sqrt{\tau_{k}\eta \left(\exp\left(\mu \tau_{k}\right)-1\right)/\mu }\right\},\\ \; & ℵ=A^{T}A+L_{1}^{2}B^{T}B-\eta I_{n}. \end{array}$$

*Then, the leader-following MAS *(1) *and* (2) *can realize the exponential consensus under the ETIM *(5) *and impulsive controllers* (3)*. Meanwhile, the Zeno behavior can be excluded.*

**Proof.**  Choose the candidate Lyapunov function

$$\begin{array}{c} V\left(e\left(t\right)\right)=e^{T}\left(t\right)e\left(t\right), \end{array}$$

whose derivative along system (4) for any $t\in \left[d_{k-1},d_{k}\right),k\in N^{+}$ is given by

$$\begin{array}{cc} D^{+}V\left(e\left(t\right)\right) & =2e^{T}\left(t\right)\dot{e}\left(t\right)\\ & =2e^{T}\left(\left(I_{N}⊗A\right)e\left(t\right)+\left(I_{N}⊗B\right)G\left(e\left(t\right)\right)\right)\\ & \leq e^{T}\left(t\right)\left(I_{N}⊗\left(A^{T}+A+R_{1}\right)\right)e\left(t\right)+G^{T}\left(e\left(t\right)\right)\left(I_{N}⊗B^{T}R_{1}^{-1}B\right)G\left(e\left(t\right)\right)\\ & \leq e^{T}\left(t\right)\left(I_{N}⊗\left(A^{T}+A+R_{1}+L_{1}^{2}B^{T}R_{1}^{-1}B\right)\right)e\left(t\right), \end{array}$$

where Lemma 1 and Assumption 1 are used in the first and the second inequalities, respectively. Therefore, it follows from (12) that for any $\left[d_{k-1},d_{k}\right),k\in N^{+}$

(17) $$D^{+}V\left(e\left(t\right)\right)\leq \mu V\left(e\left(t\right)\right).$$

On the other hand, when $t=d_{k},k\in N^{+}$, it follows from (4) that

(18) $$\begin{array}{cc} ∥e\left(d_{k}\right)∥\; & =∥∆e\left(d_{k}\right)+e\left(d_{k}^{-}\right)∥\\ \; & =∥e\left(t_{k}\right)+∆e\left(d_{k}\right)+\int_{t_{k}}^{d_{k}}\dot{e}\left(s\right)ds∥\\ \; & \leq ∥\left(H⊗K+I_{N}⊗I_{n}\right)e\left(t_{k}\right)∥+\sqrt{\tau_{k}}{\left(\int_{t_{k}}^{d_{k}}{∥\dot{e}\left(s\right)∥}^{2}ds\right)}^{\frac{1}{2}}, \end{array}$$

the last inequality can be calculated from the Schwarz inequality.

Moreover, $\left|\right|\dot{e}\left(t\right){\left|\right|}^{2}$ can be estimated by the following inequality:

(19) $$\begin{array}{cc} \; & ∥\dot{e}{\left(t\right)∥}^{2}\\ = & {\left(\left(I_{N}⊗A\right)e\left(t\right)+\left(I_{N}⊗B\right)G\left(e\left(t\right)\right)\right)}^{T}\cdot \left(\left(I_{N}⊗A\right)e\left(t\right)+\left(I_{N}⊗B\right)G\left(e\left(t\right)\right)\right)\\ = & e^{T}\left(t\right)\left(I_{N}⊗A^{T}A\right)e\left(t\right)+e^{T}\left(t\right)\left(I_{N}⊗A^{T}B\right)G\left(e\left(t\right)\right)\\ \; & +G^{T}\left(e\left(t\right)\right)\left(I_{N}⊗B^{T}A\right)e\left(t\right)+G^{T}\left(e\left(t\right)\right)\left(I_{N}⊗B^{T}B\right)G\left(e\left(t\right)\right)\\ \leq & e^{T}\left(t\right)\left(I_{N}⊗\left(A^{T}\left(I_{n}+R_{2}\right)A\right)\right)e\left(t\right)+G^{T}\left(e\left(t\right)\right)\left(I_{N}⊗\left(B^{T}\left(I_{n}+R_{2}^{-1}\right)B\right)\right)G\left(e\left(t\right)\right)\\ \leq & e^{T}\left(t\right)\left(I_{N}⊗\left(A^{T}\left(I_{n}+R_{2}\right)A+L_{1}^{2}B^{T}\left(I_{n}+R_{2}^{-1}\right)B\right)\right)e\left(t\right)\\ \leq & {\eta ∥e\left(t\right)∥}^{2}, \end{array}$$

where the last inequality is obtained from (13), and when $t\in [d_{k-1},d_{k})$. It follows from (17) that $V\left(e\left(t\right)\right)\leq \exp\left(\mu \left(t-t_{k}\right)\right)V\left(e\left(t_{k}\right)\right)$, for any $t\in [t_{k},d_{k})$, $k\in N^{+}$. Thus, we have from (19)

(20) $$\begin{array}{cc} {∥\dot{e}\left(t\right)∥}^{2} & \leq \eta \exp\left(\mu \left(t-t_{k}\right)\right){∥e(t_{k})∥}^{2}. \end{array}$$

Substituting (20) into (18), it follows from (14) that

(21) $$\begin{array}{cc} \; & \;\;∥e(d_{k})∥\\ \; & \leq \sqrt{\tau_{k}}{\left(\int_{t_{k}}^{d_{k}}\eta {∥e\left(t_{k}\right)∥}^{2}\exp\left(\mu \left(s-t_{k}\right)\right)ds\right)}^{\frac{1}{2}}+∥\left(H⊗K+I_{Nn}\right)e\left(t_{k}\right)∥\\ \; & \leq \sqrt{\tau_{k}}{\left(\int_{t_{k}}^{d_{k}}\eta {∥e\left(t_{k}\right)∥}^{2}\exp\left(\mu \left(s-t_{k}\right)\right)ds\right)}^{\frac{1}{2}}+\sqrt{\rho_{0}}∥e\left(t_{k}\right)∥\\ \; & \leq \rho ∥e\left(t_{k}\right)∥. \end{array}$$

By further simplifying (21), the following inequality holds when $t=d_{k},k\in N^{+}$,

(22) $$V\left(e\left(d_{k}\right)\right)\leq \rho^{2}V\left(e\left(t_{k}\right)\right).$$

Therefore, based on inequalities (5) and (22), the following facts can be obtained.

There is $V\left(e\left(t\right)\right)\leq a\exp\left(-b\left(t-t_{0}\right)+\left(t-t_{1}\right)\left(\mu +b\right)\right)$ for $t\in [d_{0},d_{1})$, and $t=d_{1}$ has

(23) $$\begin{array}{cc} V(e\left(d_{1}\right))\; & \leq \rho^{2}V\left(e\left(t_{1}\right)\right)=a\rho^{2}\exp(-b\left(d_{1}-t_{0}\right)\\ \; & \leq a\rho^{2}\exp\left(-b\left(d_{1}-t_{0}\right)+\tau_{1}\left(\mu +b\right)\right). \end{array}$$

There is $V\left(e\left(t\right)\right)\leq a\rho^{2}\exp\left(-b\left(t-t_{0}\right)+\left(\tau_{1}+t-t_{2}\right)\left(\mu +b\right)\right)$ for $t\in [d_{1},d_{2})$, and $t=d_{2}$ has

$$V\left(e\left(d_{2}\right)\right)\leq a\rho^{4}\exp\left(-b\left(d_{2}-t_{0}\right)+\sum_{i=1}^{2}\tau_{i}\left(\mu +b\right)\right).$$

There is $V\left(e\left(t\right)\right)\leq a\rho^{4}\exp\left(-b\left(t-t_{0}\right)+\left(\sum_{i=1}^{2}\tau_{i}+t-t_{3}\right)\left(\mu +b\right)\right)$ for $t\in [d_{2},d_{3})$, and $t=d_{3}$ has

$$V\left(e\left(d_{3}\right)\right)\leq a\rho^{6}\exp\left(-b\left(d_{3}-t_{0}\right)+\sum_{i=1}^{3}\tau_{i}\left(\mu +b\right)\right).$$

By the Mathematical Induction Method, the following inequality holds for any $t\in \left[d_{k-1},d_{k}\right),k\in N^{+}$:

(24) $$\begin{array}{c} V\left(e\left(t\right)\right)\leq a\rho^{2(k-1)}\exp\left(-b\left(t-t_{0}\right)+(\sum_{i=1}^{k-1}\tau_{i}+t-t_{k})\left(\mu +b\right)\right), \end{array}$$

where $\sum_{i=1}^{0}=0$ is used. Furthermore, by using (15), one has

(25) $$\begin{array}{c} V\left(e\left(t\right)\right)\leq \frac{a}{\rho^{2}}\exp\left(-b\left(t-t_{0}\right)\right). \end{array}$$

Hence, (25) implies that system (1) and (2) can achieve the exponential consensus, since $a>V(e\left(t_{0}\right))$, and thus V(e(t))→0 as t→+∞. Moreover, conditions in Theorem 1 are satisfied (see (17) and (22)), and Zeno behavior can be excluded at the same time, i.e.,

$$\underset{k\in N^{+}}{\inf}\left\{t_{k+1}-t_{k}\right\}\geq -\frac{2}{b+\mu }\ln\rho .$$

Thus, the proof of Theorem 2 is completed. □

**Remark 5.**  *Note that forced impulses are usually used to ensure the exponential consensus, and they are not required here, since the threshold is already decaying with an exponential rate. Moreover, the conditions are more easy to check compared with the results in [33], and this mainly relies on the advantage of using impulsive control.*

**Remark 6.**  *To ensure exponential stability of system *(4)*, the actuation delay must be satisfied that $\rho^{2}\exp\left(\tau_{k}\left(\mu +b\right)\right)<1,\forall k\in N^{+}$, i.e., $\sqrt{\tau_{k}\eta \mu^{-1}\left(\exp\left(\mu \tau_{k}\right)-1\right)\exp\left(\tau_{k}\left(\mu +b\right)\right)}<1-\sqrt{\rho_{0}\exp\left(\tau_{k}\left(\mu +b\right)\right)}$. However, this inequality is transcendental, making it difficult to verify the parameter $\tau_{k}$ directly. To simplify the verification, we assume that $\tau_{k}<\mu /\eta$ and $\rho_{0}\in \left(0,\frac{1}{2}\right)$. Then, a sufficient condition to ensure the above inequality holds that $\exp\left(\tau_{k}\left(2\mu +b\right)\right)<2+2\rho_{0}\exp\left(\tau \left(\mu +b\right)\right)$, i.e, $\tau_{k}<\ln\frac{2}{1-2\rho_{0}}/\left(2\mu +b\right)$. It is worth noting that actuation delays have a negative impact on the stability of the system, as increasing the delay makes the inequality $\rho^{2}\exp\left(\tau_{k}\left(\mu +b\right)\right)$ no longer valid.*

**Remark 7.**  *Let $N(t,t_{0})$ represent the number of impulses in the interval $\left(t_{0},t\right)$ and if an impulsive sequence adopts an average impulsive interval h, then $\frac{t-t_{0}}{h}-N_{0}\leq N\left(t,t_{0}\right)\leq \frac{t-t_{0}}{h}+N_{0}$, where $N_{0}>0$. Compared with the event-triggered impulsive control, the frequency of impulses in traditional impulsive control (time-triggered) needs to be fast enough, i.e., $\frac{1}{h}$ bigger than a constant. Considering the traditional impulsive control, under which system *(1) *and* (2) *can achieve the exponential consensus if the following condition is satisfied: h∈(0,−2lnρ), since it follows from *(17) *and* (22)* that*

(26) $$\begin{array}{cc} V(e(t))\; & \leq \rho^{2k}\exp\left(\mu \left(t-t_{0}\right)\right)V\left(e\left(t_{0}\right)\right)\\ \; & \leq \rho^{2}\exp\left(\frac{2\left(t-t_{0}\right)}{h}\ln\rho +\mu \left(t-t_{0}\right)\right)V\left(e\left(t_{0}\right)\right)\\ \; & \leq \rho^{2}\exp\left(\theta \left(t-t_{0}\right)\right)V\left(e\left(t_{0}\right)\right), \end{array}$$

*where θ=2lnρ/h+μ<0.*

**Remark 8.**  *Consider the event-triggered mechanism *(5) *with continuous control, where*

$$u_{i}\left(t\right)=K_{c}\sum_{j\in N_{i}}\left[a_{ij}\left(x_{i}\left(t-\tau_{k}\right)-x_{j}\left(t-\tau_{k}\right)\right)+c_{i}\left(x_{i}\left(t-\tau_{k}\right)-x_{0}\left(t-\tau_{k}\right)\right)\right],$$

*with $K_{c}\in R^{n\times n}$. Then, the error system *(4) *can be rewritten as*

$$\dot{e}\left(t\right)=\left(I_{N}⊗A+H⊗K_{c}\right)e\left(t\right)+\left(I_{N}⊗B\right)G\left(e\left(t\right)\right),t\geq t_{0},$$

*and the derivative along it for any $t\in \left[d_{k-1},d_{k}\right),k\in N^{+}$ is given by*

$$\begin{array}{cc} D^{+}V\left(e\left(t\right)\right)\; & =2e^{T}\left(\left(I_{N}⊗A+H⊗K_{c}\right)e\left(t\right)+\left(I_{N}⊗B\right)G\left(e\left(t\right)\right)\right)\\ \; & \leq e^{T}\left(t\right)(I_{N}⊗\left({\left(A+H⊗K_{c}\right)}^{T}+A+H⊗K_{c}+R_{1}+L_{1}^{2}B^{T}R_{1}^{-1}B\right))e\left(t\right)\\ \; & \leq -\bar{\mu }V\left(e\left(t\right)\right), \end{array}$$

*where $\bar{\mu }>0$ such that*

(27) $$\left[\begin{array}{cc} I_{N}⊗\left({\left(A+H⊗K_{c}\right)}^{T}+A+H⊗K_{c}+R_{1}+\bar{\mu }I_{n}\right) & L_{1}\left(I_{N}⊗B^{T}\right)\\ {}* & -I_{N}⊗R_{1} \end{array}\right]<0,$$

*and it indicates that system *(1) *and* (2) *can achieve the exponential consensus under the event-triggered mechanism *(5)* with continuous control.*

In addition, if the actuation delay is constant, i.e., $\tau_{k}\equiv \tau$, then according to Remark 6, the result can be obtained as Corollary 1, whose proof is similar to the one given for Theorem 2.

**Corollary 1.**  *Suppose that $\tau_{k}\equiv \tau \geq 0$. Then, Zeno behavior can be excluded, and system *(1) *and* (2) *can realize the exponential consensus under the ETIM *(5) *and impulsive controllers* (3)*, if conditions in Theorem 2 hold except *(16)*, $\rho_{0}\in \left(0,\frac{1}{2}\right)$, and*

$$\tau <\min\left\{\frac{\mu }{\eta },\frac{1}{\left(2\mu +b\right)}\ln\frac{2}{1-2\rho_{0}}\right\}.$$

## 4. Numerical Example

**Figure 2:**
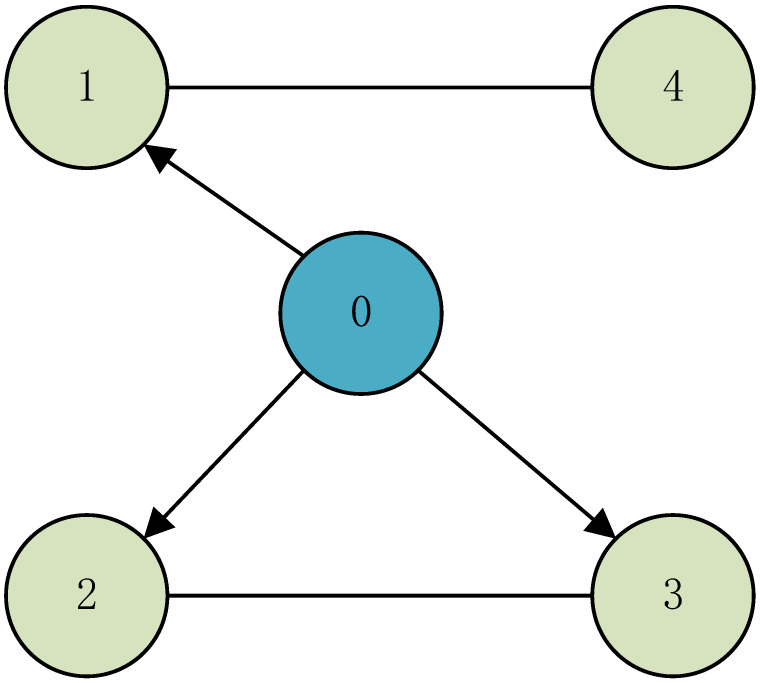
The communication topology.

Consider a three-dimensional leader-following MAS with one leader and four followers, whose dynamics are described in system (1) and (2), respectively. The communication topology is shown, from which one can check that Figure 2.

$$L=\left[\begin{array}{cccc} 1 & 0 & 0 & -1\\ 0 & 1 & -1 & 0\\ 0 & -1 & 1 & 0\\ -1 & 0 & 0 & 1 \end{array}\right],\;C=\left[\begin{array}{cccc} 1 & 0 & 0 & 0\\ 0 & 1 & 0 & 0\\ 0 & 0 & 1 & 0\\ 0 & 0 & 0 & 0 \end{array}\right],\;H=\left[\begin{array}{cccc} 2 & 0 & 0 & -1\\ 0 & 2 & -1 & 0\\ 0 & -1 & 2 & 0\\ -1 & 0 & 0 & 1 \end{array}\right].$$

For any $l\in \left\{0,1,2,3,4\right\}$, let $f\left(x_{l}\left(t\right)\right)=\tanh\left(x_{l}\left(t\right)\right)$, then we have $L_{1}=1$. Furthermore, choose the following constant matrices:

$$A=\left[\begin{array}{ccc} 0.67 & 0 & 0\\ 1 & 1 & 1\\ 0 & -0.32 & 0 \end{array}\right],\;\;B=\left[\begin{array}{ccc} 0.51 & 0 & 0\\ 0 & 0.42 & 0\\ 0 & 0.21 & 0.45 \end{array}\right].$$

By selecting μ=2.5, η=0.3, $K\equiv -0.45I_{3}$. The parameters in conditions (12) and (13) can be solved by LMIs, $\rho_{0}=0.6175$ and

$$R_{1}=\left[\begin{array}{ccc} 1.92 & -0.5 & 0\\ -0.5 & 2.25 & -0.34\\ 0 & -0.34 & 1.25 \end{array}\right],\;R_{2}=\left[\begin{array}{ccc} 2.5522 & 0.1028 & 0.2046\\ 0.1028 & 1.5437 & -0.8068\\ 0.2046 & -0.8068 & 2.5764 \end{array}\right].$$

Then, we have ρ=0.6458 and $\tau_{\sup}<0.0566$. Choose a random actuation delay $\tau_{k}$ at each event-triggered instants according to the distribution $\tau_{k}=0.01*\mathrm{randi}\left([1,5],1,1\right)$, where randi([1,5],1,1) denotes the random selection of one element from the set {1,2,3,4,5}. In addition, let $t_{0}=0$ and initial states be randomly selected as

$$\begin{array}{cc} {}[x_{0}\left(t_{0}\right), & x_{1}\left(t_{0}\right),x_{2}\left(t_{0}\right),x_{3}\left(t_{0}\right),x_{4}\left(t_{0}\right)]=\\ & \left[\begin{array}{ccccc} 4.5 & 5.5 & -1.5 & -0.2 & 1\\ -3.2 & 2.5 & -1 & -2 & -1.5\\ 2 & -2.5 & 0.5 & 2.8 & 0.2 \end{array}\right]. \end{array}$$

**Figure 3:**
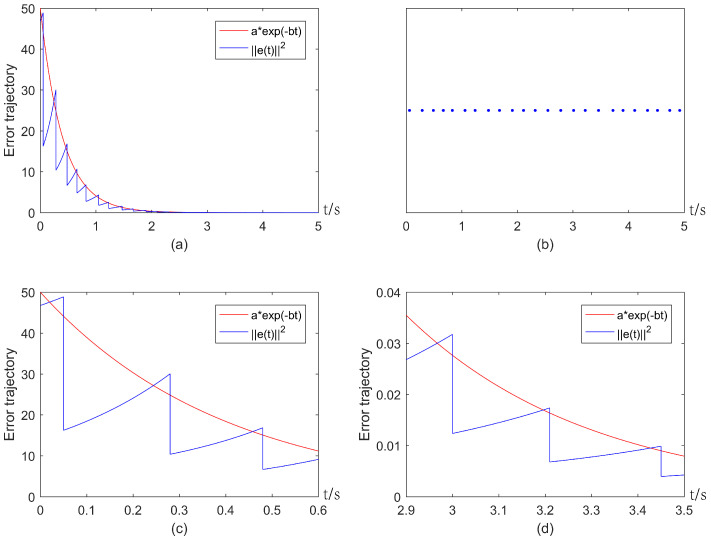
Exponential consensus under impulsive control (3) and the ETIM (5) with actuation delays: (**a**) Error trajectory of system; (**b**) Triggering instants generated by the ETIM (5); (**c**) Local part of (**a**) in the interval [0,0.6]; (**d**) Local part of (**a**) in the interval [2.9,3.5].

By setting a=50 (since $V(e\left(t_{0}\right))=46.75$), and b=2.5, one can verify that the conditions in Theorems 1 and 2 are satisfied. The threshold aexp(−bt) and trajectories of error states under impulsive control (3) and the event-triggered mechanism (5) are depicted in Figure 3a. Figure 3a shows that ${\left||e\left(t\right)|\right|}^{2}$ will exceed the threshold at each event-triggered instant caused by actuation delays, and trajectories of error states finally converge to zero, i.e., exponential consensus can be reached. The corresponding event-triggered instants are shown in Figure 3b. Meanwhile, some local parts of Figure 3a are depicted in Figure 3c,d, which shows that Zeno behavior is avoided.

**Figure 4:**
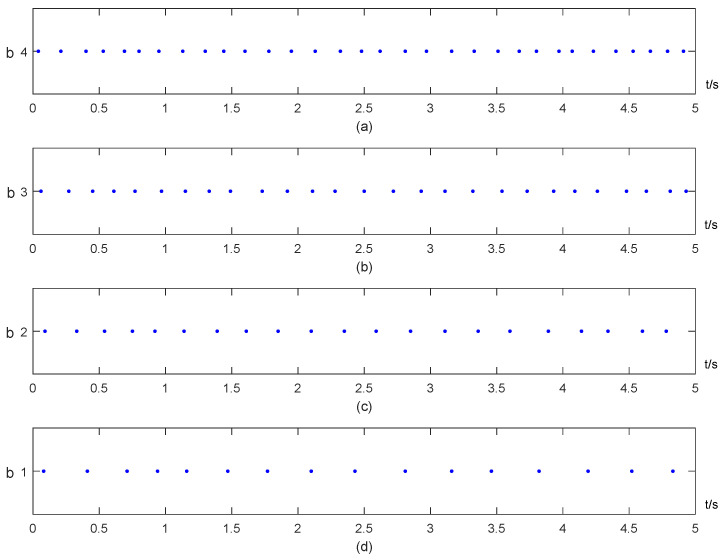
Triggering instants of ETIM (5) with different b: (**a**) *b* = 4; (**b**) *b* = 3; (**c**) *b* = 2; (**d**) *b* = 1.

To describe the triggering parameters in the ETIM (5), we fixed other parameters as selected above except *b*. The simulations with different *b* are shown in Figure 4. It is obvious from Figure 4 that a larger *b* will generate more triggering instants (see Figure 4a–d), i.e., it leads to a lower event trigger interval; and this coincides with (11). Furthermore, the effect of the parameter *a* is not obvious since it is independent of (11).

**Figure 5:**
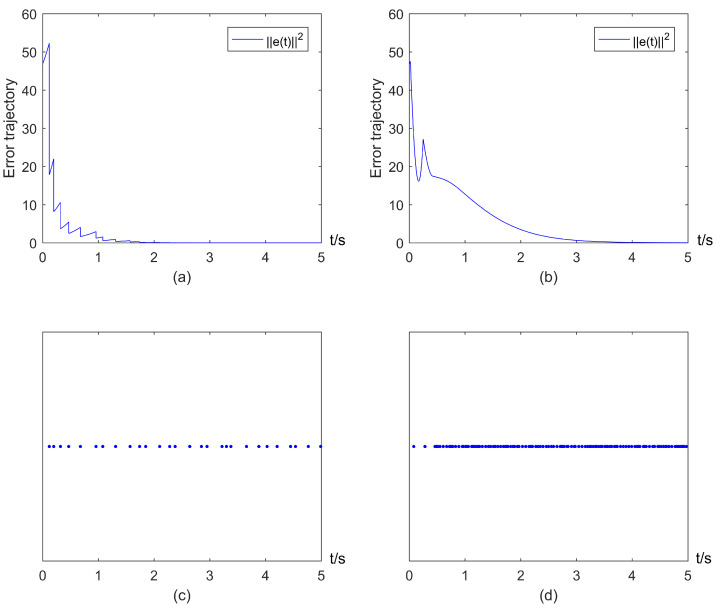
Exponential consensus under traditional impulsive control and event-triggered control: (**a**) Error trajectory of system under traditional impulsive control; (**b**) Error trajectory of system under continuous-time event-triggered control; (**c**) Impulsive instants with h=0.2; (**d**) Triggering instants generated by the event-triggered mechanism (5).

To highlight the designed ETIM (5), we further consider the traditional impulsive control and continuous-time event-triggered control, and the conditions to ensure exponential consensus are given in Remarks 7 and 8, respectively. Choosing $K=-0.45I_{3},h=0.2\in \left(0,0.3499\right)$ and $K_{c}=-4.5I_{3}$ (by solving LMI (27) with $\bar{\mu }=0.1$), the simulations are depicted in Figure 5.

From Figure 5a,b, one can conclude that the exponential consensus can be reached with impulsive control and event-triggered control, respectively. The corresponding impulsive/triggering instants are given, respectively, in Figure 5c,d. It can be observed that the numbers of impulsive instants are bigger than those depicted in Figure 3b, while triggering instants generated by continuous-time event-triggered control are much more than those in Figure 3b. Hence, the performance of the designed ETIM (5) is better than the traditional impulsive control and continuous-time event-triggered control.

**Figure 6:**
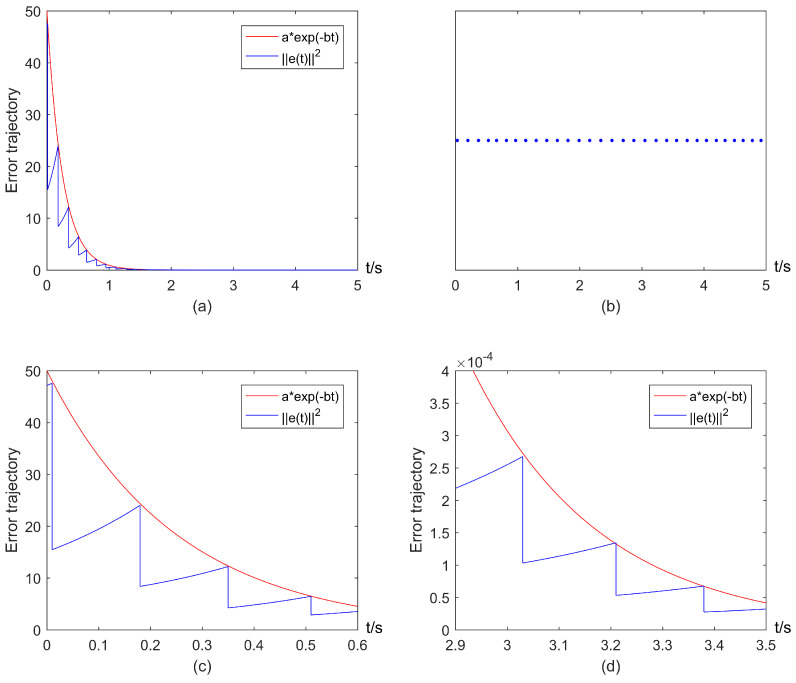
Exponential consensus under impulsive control (3) and the ETIM (5) without actuation delays: (**a**) Error trajectory of system; (**b**) Triggering instants generated by the ETIM (5); (**c**) Local part of (**a**) in the interval [0,0.6]; (**d**) Local part of (**a**) in the interval [2.9,3.5].

In addition, consider the special case with $\tau_{k}\equiv 0$, and the corresponding error trajectories and threshold aexp(−bt) for this case are shown in Figure 6; event-triggered instants shown in Figure 6. Similarly, it can be concluded that exponential consensus can be reached without exhibiting Zeno behavior.

## 5. Conclusions

In this paper, an event-triggered impulsive control method subject to actuation delays is proposed for a class of nonlinear leader-following MASs to achieve exponential consensus. Under the designed ETIM, Zeno behavior is shown to be excluded, and to realize exponential consensus, sufficient conditions relating to actuation delays and impulsive strength, are also established. However, it should be noted that the ETIM designed in this paper is susceptible to external disturbances, so designing an anti-disturbance strategy is an important consideration. Additionally, it would be worthwhile to extend these results to dynamic ETIMs, as event-triggered instants can be further reduced in such cases.

## Data Availability

Not applicable.
